# Supplementary figures and images for: HIF drives lipid deposition and cancer in ccRCC via repression of fatty acid metabolism
Source: Nat Commun. 2017 Nov 24;8:1769. doi: 10.1038/s41467-017-01965-8 (PMC5701259; doi:10.1038/s41467-017-01965-8)

Supplementary figure 1: Uncropped blots.

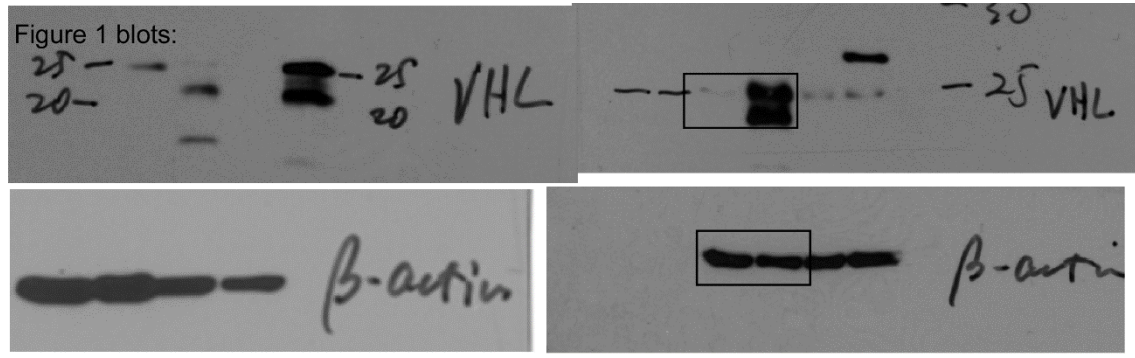

Figure 4 blots:

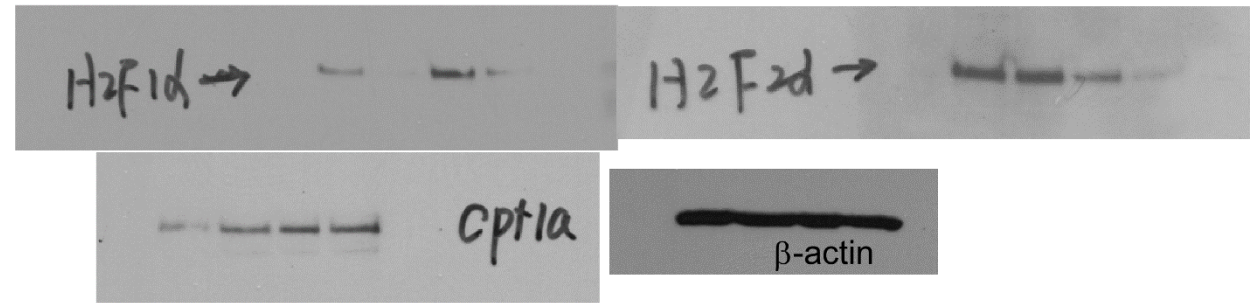

Figure 5 blots:

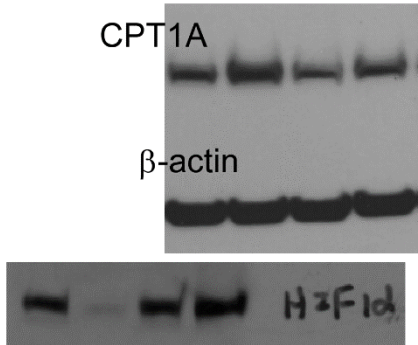

Figure 6 blots:

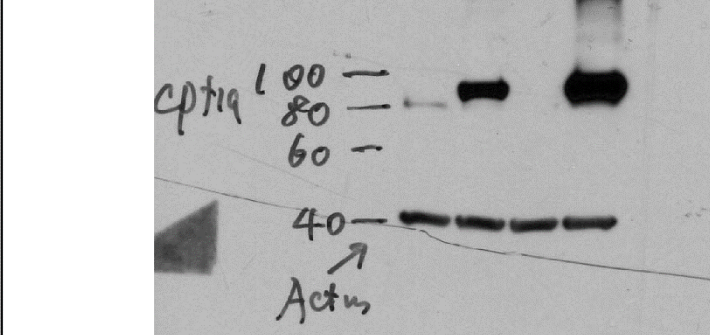

Figure 7 blots:

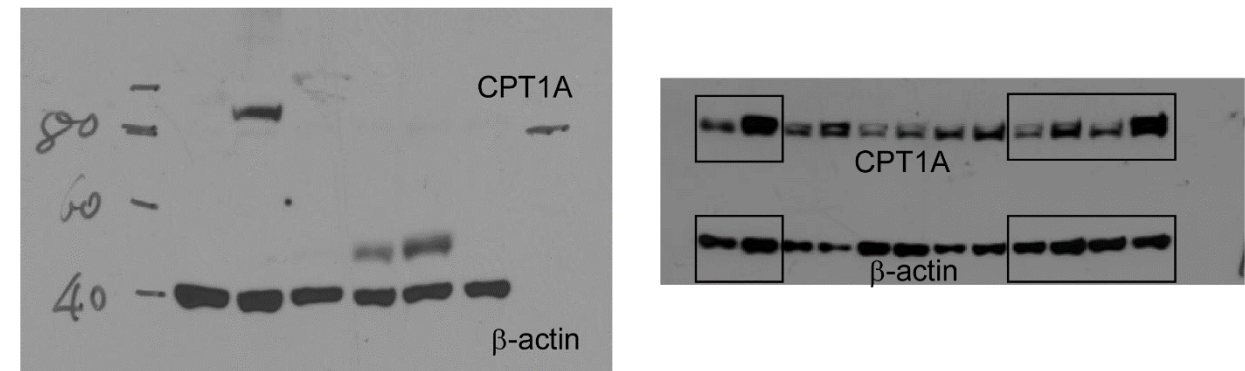

Supplement: Supplementary file 1 — Supplementary Information [file 41467_2017_1965_MOESM1_ESM.pdf]
